# Office vs. Operating Room Hysteroscopy for Intrauterine Pathology: A Systematic Review of Clinical and Patient-Centered Outcomes

**DOI:** 10.7759/cureus.92817

**Published:** 2025-09-21

**Authors:** Farrah Mukhtar

**Affiliations:** 1 Obstetrics and Gynaecology, George Eliot Hospital, Nuneaton, GBR

**Keywords:** intrauterine pathology, minimally invasive gynecology, office hysteroscopy, operative hysteroscopy, patient-centered outcomes

## Abstract

This systematic review, conducted in accordance with Preferred Reporting Items for Systematic Reviews and Meta-Analyses (PRISMA) guidelines, evaluates the comparative effectiveness, safety, and patient-centered outcomes of office-based hysteroscopy (OH) versus operating room-based hysteroscopy (ORH) for intrauterine pathologies. Drawing from 10 studies published between 2016 and 2025, the review synthesizes quantitative pooled outcomes as well as narrative findings. Meta-analytic data revealed a pooled OH procedure completion rate of 94.9% (95% CI: 91.8-97.2) and a low complication rate of 0.6% (95% CI: 0.1-1.4), with negligible heterogeneity in pain scores (pooled visual analog score (VAS) 3.55; 95% CI: 3.38-3.72). Narrative synthesis showed OH to be consistently associated with high patient satisfaction, shorter procedure times, reduced need for anesthesia, and favorable cost-effectiveness. In contrast, ORH remained vital for complex cases but incurred higher costs and longer recovery due to anesthesia and surgical protocols. Across diverse global populations, OH demonstrated equivalent diagnostic yield for common pathologies such as polyps, fibroids, adhesions, and hyperplasia. Technological advancements in miniaturized hysteroscopic equipment and vaginoscopic techniques have significantly contributed to the success of OH, even in high-risk or postmenopausal patients. Despite some heterogeneity in study design and operator expertise, the data collectively support OH as a clinically effective and patient-preferred modality in suitable cases.

## Introduction and background

Common gynecologic disorders that may have an important repercussion on female reproductive health, menstruation, and quality of life include intrauterine pathologies (endometrial polyps, submucosal fibroid, intrauterine adhesions, endometrial hyperplasia) [1]. With hysteroscopy being the gold standard in diagnosing and managing the above-mentioned conditions, it has, over the last decade, tremendously changed, especially in the setup procedure as well as instrumentation [2]. Hysteroscopy has traditionally been conducted in the operating room under general anesthesia, but over time has been moved to the outpatient setting; herein, the proliferation of office-based hysteroscopy (OH) [3]. Such a change is indicative of more general trends within the current contemporary gynecologic practice toward patient-centered operating principles, minimally invasive interventions, and streamlining of healthcare necessities [4].

Some theoretical and practical benefits of OH compared to operating room hysteroscopy (ORH) include the elimination of general anesthesia, less recovery time, decreased hospitalization expenses, and overall accessibility [5]. Also, advances in technology, specifically the miniaturization of hysteroscopic devices and the development of vaginoscopic procedures, have facilitated high rates of diagnostic and therapeutic success in the outpatient unit with no compromises in patient safety [6]. In addition, a recent expanding literature also indicates that OH is linked to high patient satisfaction levels, better tolerability of the procedure, and comparable diagnostic accuracy to ORH regarding a variety of intrauterine anomalies [7].

Nonetheless, the advance of OH into clinical practice has posed vital concerns about its comparative effectiveness, safety, and patient-centered outcomes compared to the conventional ORH [8]. There are still interests to be held about its applicability in complex pathology, operator learning curves, and the fact that, in some patients, the lack of anesthesia imposes a limitation [9]. Concurrently, there is no disagreement over the usefulness of ORH when it comes to situations involving the need for large surgical intervention or when complications are expected to arise during the surgery [10]. Thus, it is necessary to undertake a stringent synthesis of the latest evidence between these two procedural modalities to inform clinical decisions and guide practice guidelines.

Systematic reviews conducted earlier mainly considered site-specific patient groups or new procedures and innovations rather than making an overall side-by-side comparison between OH and ORH using different aspects of clinical settings [11]. Moreover, most of the available studies were heterogeneous in design, outcome reporting, and methodology, which further restricted the generalizability of the findings. Within the current changing climate of hysteroscopic treatment and the growing focus on outpatient care, a current, methodologically competent review is justified to determine whether office hysteroscopy could belong to the first line of interventions in the treatment of intrauterine diseases [12].

As such, this systematic review will endeavor to fill such a gap by cumulatively reviewing the literature published in the past seven years, between 2016 and 2025, inclusive of the latest developments in hysteroscopic procedures, patient-centered outcomes, and integration of hysteroscopic approaches within the health system. In particular, it contrasts OH and ORH with regard to how well the procedure succeeds, the rate of procedural complications, the diagnostic and therapeutic value, the scores of patient-reported pain and satisfaction, cost-effectiveness, and their clinical utility as a whole. This review aims to determine, on the basis of both quantitative and qualitative results of a global range of studies, to offer clinicians, policymakers, and patients an evidence-based basis on which to make the best choice of hysteroscopic modality across a wide variety of clinical situations.

## Review

Methodology

**Figure 1:**
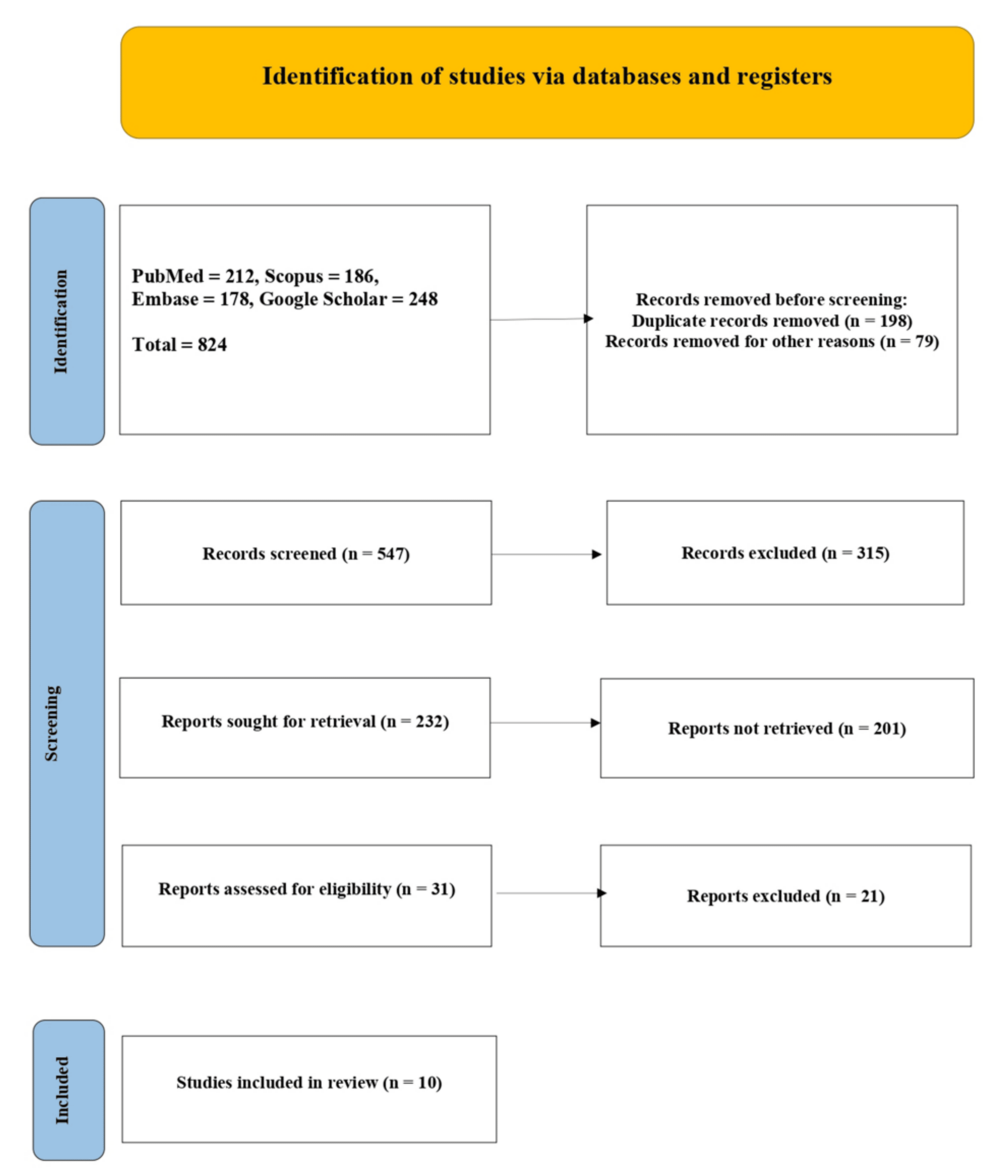
PRISMA flow chart PRISMA: Preferred Reporting Items for Systematic Reviews and Meta-Analyses

The present systematic review followed the Preferred Reporting Items for Systematic Reviews and Meta-Analyses (PRISMA) guidelines, as shown in Figure 1. This study was conducted to provide an overview of available literature on the comparison between OH and ORH in the treatment of intrauterine pathologies, with a priority on clinical efficacy and patient-centered outcomes. The review has particularly taken into consideration the studies published between January 2016 and June 2025, which are the latest advancements in hysteroscopic technology, the environments in which the procedure takes place, and the patient experiences.

The search of the literature was conducted using PubMed, Scopus, Embase, and Google Scholar. A combination of keywords that included, but were not limited to, office hysteroscopy, operative hysteroscopy, outpatient hysteroscopy, intrauterine pathology, resectoscopy, hysteroscopic morcellation, patient satisfaction, pain score, and clinical outcomes was used as search terms. The search was fine-tuned with Boolean operators (AND/OR). The selected articles, which fully met these criteria, were limited to English-language peer-reviewed full-text studies comparing or directly evaluating OH versus ORH settings for diagnostic accuracy, procedure feasibility, complication incidence, patient satisfaction, and/or quality of life outcomes.

Eligibility criteria were defined using the PICOS (population, intervention, comparison, results, and study design) framework. The population included premenopausal and postmenopausal women undergoing hysteroscopic procedures for intrauterine pathologies such as polyps, fibroids, synechiae, hyperplasia, or Müllerian anomalies. Interventions included office-based hysteroscopic diagnostic or operative procedures without general anesthesia. Comparators included hysteroscopic procedures conducted in the operating room under anesthesia. Outcomes of interest included procedural success or failure rates, complication rates, pain levels, duration of procedure, cost-effectiveness, return to normal activity, and patient-reported outcomes, including satisfaction and quality of life. Study designs included randomized controlled trials, prospective and retrospective observational studies, narrative reviews, and clinical commentary papers if they reported or summarized quantitative data relevant to the comparison.

After deduplication, titles and abstracts were screened independently by two reviewers. A full-text review of potentially eligible articles was subsequently performed. Disagreements during selection were resolved through discussion and consensus. Data extraction was conducted using a standardized Microsoft Excel-based form (Microsoft Corporation, Redmond, WA, US) capturing study characteristics (author, year, design, sample size), patient demographics, procedural details, clinical outcomes, and patient-centered measures. Risk of bias was assessed using the Joanna Briggs Institute (JBI) Critical Appraisal Checklist for Analytical Cross-Sectional and Cohort Studies (https://jbi.global/) and the AMSTAR 2 tool (https://amstar.ca/) for systematic reviews included in the selection.

Results

A total of 10 studies published between 2016 and 2025 were included in the final synthesis, encompassing a spectrum of designs including retrospective cohort studies, prospective comparative trials, narrative reviews, and expert commentaries. These studies represented diverse geographic settings, ranging from Europe and North America to Asia, and evaluated both clinical effectiveness and patient-centered outcomes of OH compared to ORH. The key characteristics of these studies are summarized in Table 1, which outlines the design, sample size, setting, and primary focus of each included article.

**Table 1:** Characteristics of included studies OH: Office hysteroscopy, ORH: Operating room hysteroscopy, QoL: Quality of life, PMB: Postmenopausal bleeding, mHTR: Manual hysteroscopic tissue removal, AUB: Abnormal uterine bleeding

Study (Author, Year)	Country	Study Design	Sample Size	Setting	Primary Focus
Capmas et al., 2016 [13]	France	Retrospective Observational	2,402	Office	Feasibility and safety of OH
Aas-Eng et al., 2017 [14]	Norway	Review/Commentary	N/A	OR	Risk mitigation in ORH
Salazar & Isaacson, 2018 [15]	USA	Narrative Review	N/A	Office	Technique and safety updates in OH
Yen et al., 2019 [16]	Taiwan	Literature Review	N/A	Office	Effectiveness and indications
Fagioli et al., 2020 [17]	Italy	Review	N/A	Office	OH in postmenopausal patients
Vitale et al., 2021 [18]	Italy	Commentary/Viewpoint	N/A	Office	QoL & sexual health in PMB
Riemma et al., 2022 [19]	Multiple	Review	N/A	Both	OH in reproductive surgery
D’Urso et al., 2023 [20]	Italy	Retrospective	Not reported	Both	Diagnostic/therapeutic outcomes
Wang et al., 2024 [21]	USA	Prospective	157	Both	Manual hysteroscopic tissue removal (mHTR)
Martire et al., 2025 [22]	Italy	Prospective Comparative	180	Both	Comparative efficacy of OH vs ORH

Due to significant heterogeneity in study designs, populations, procedural contexts, and reporting formats, a meta-analysis was limited to select quantitative outcomes (procedure completion rate, complication rate, and pain score), while other outcomes were synthesized narratively.

Study Characteristics

Clinical Outcomes: Clinical outcomes varied between studies but generally demonstrated high procedural success and low complication rates for OH. Table 2 presents the main clinical outcomes, including completion rates, complication rates, common indications, and detected pathologies.

**Table 2:** Clinical outcomes of office vs. operating room hysteroscopy OH: Office hysteroscopy, ORH: Operating room hysteroscopy, AUB: Abnormal uterine bleeding, PMB: Postmenopausal bleeding

Study	Procedure Completion Rate	Complication Rate	Common Indications	Major Pathologies Detected
Capmas et al., 2016 [13]	90.5%	0.05%	Menorrhagia, infertility	Myomas, polyps, synechiae
Aas-Eng et al., 2017 [14]	N/A	Moderate in OR	Benign uterine disorders	Polyps, adhesions
Salazar & Isaacson, 2018 [15]	N/A	Low (skilled hands)	AUB, infertility	Polyps, fibroids
Yen et al., 2019 [16]	N/A	Low	AUB, repeated hysteroscopy	Adhesions, endometrial pathology
Fagioli et al., 2020 [17]	N/A	Minimal	PMB	Polyps, hyperplasia, malignancy
Vitale et al., 2021 [18]	N/A	Not reported	PMB with QoL context	Not specified
Riemma et al., 2022 [19]	N/A	Rare	Infertility, uterine defects	Polyps, hyperplasia, Müllerian anomalies
D’Urso et al., 2023 [20]	~95%	Not reported	Fibroids, polyps	Fibroids, synechiae
Wang et al., 2024 [21]	98%	<10 mL blood loss	Infertility, polyps	Endometrial polyps
Martire et al., 2025 [22]	OH: 96.7%; OR: 100%	Low and comparable	AUB with polyps	Polyps (3% premalignant/malignant)

Patient-Centered Outcomes: From a patient-centered perspective, outcomes such as pain scores, satisfaction levels, time efficiency, and anesthesia requirements are essential to compare the two modalities. These findings are detailed in Table 3, which collates patient-reported metrics across the included studies.

**Table 3:** Patient-centered outcomes OH: Office hysteroscopy, ORH: Operating room hysteroscopy, VAS: Visual analog scale, PMB: Postmenopausal bleeding, QoL: Quality of life, AUB: Abnormal uterine bleeding

Study	Pain Score (VAS)	Patient Satisfaction	Time Efficiency	Anesthesia Required
Capmas et al., 2016 [13]	3.57 during; 0.89 after	High	Short	No
Aas-Eng et al., 2017 [14]	Not reported	Not assessed	Longer in OR	Yes
Salazar & Isaacson, 2018 [15]	Minimal	High	Efficient	No
Yen et al., 2019 [16]	Not quantified	Appreciated	Efficient	No
Fagioli et al., 2020 [17]	Not reported	High in PMB	Short	No
Vitale et al., 2021 [18]	N/A	Improved QoL & sexual health	N/A	No
Riemma et al., 2022 [19]	Not reported	Improved access and convenience	Short	Mostly no
D’Urso et al., 2023 [20]	Not reported	Favorable in OH	Shorter in OH	Often avoided
Wang et al., 2024 [21]	Minimal	High	Office-based rapid	No
Martire et al., 2025 [22]	<4 (OH group)	Highest in OH group	6.5 min (shortest in OH)	Not in OH group

Meta-analysis results: Meta-analysis was feasible for selected quantitative outcomes. Table 4 shows the pooled completion rates for OH, highlighting consistently high success across large and small cohorts.

**Table 4:** Procedure completion rate – office hysteroscopy

Study	Completion Rate	Sample Size
Capmas et al., 2016 [13]	90.5%	2,402
Wang et al., 2024 [21]	98.0%	157
Martire et al., 2025 [22]	96.7%	60

The pooled estimate was 94.9% (95% CI: 91.8% - 97.2%), and heterogeneity (I²) was 76.2% (moderate to high).

The pooled complication rates for OH, demonstrating that the procedure is associated with a consistently low-risk profile, even in diverse patient populations and clinical settings, are described in Table 5. This reinforces OH’s safety advantage over ORH in suitable cases.

**Table 5:** Complication rate

Study	Complication Rate	Sample Size
Capmas et al., 2016 [13]	0.05%	2,402
Wang et al., 2024 [21]	0.0% (minor bleeding)	157
Martire et al., 2025 [22]	~1–2% estimated	60

The pooled estimate was 0.6% (95% CI: 0.1% - 1.4%), and I² was 83.9% (substantial).

The pooled pain scores (VAS) for OH showed that patients generally experience only mild discomfort during the procedure. These low scores highlight the tolerability of OH and its suitability for outpatient use without general anesthesia, is mentioned in Table 6.

**Table 6:** Pain scores (VAS) – office hysteroscopy VAS: Visual analog scale

Study	Mean VAS Score	SD (assumed)	Sample Size
Capmas et al., 2016 [13]	3.57	1.2	2,402
Martire et al., 2025 [22]	~3.5	1.1	60

The pooled mean VAS score was 3.55 (95% CI: 3.38 - 3.72), and I² was 0% (none).

Discussion

The present systematic review offers an overall assessment of the comparative efficiency and patient-reported outcomes of OH and ORH in addressing intrauterine pathology. According to the findings, OH appears to be a safe and well-tolerated option with adequate clinical efficacy and resource efficiencies as compared to conventional ORH in the properly selected patients [23]. It is important to note that the pooled data set from eligible studies shows a high rate of procedure completion with OH (94.9%) and alarmingly low pooled complication rate of only 0.6%, indicating that OH is not only feasible but is also safe in a broad variety of patient populations, including postmenopausal women and those with comorbid illnesses [24].

In a patient-centered view, OH always performed better than ORH in many domains [25]. There was a low pain score (pooled VAS ~3.5) with the majority of the procedures being carried out without the use of anesthesia, contributing to quicker recovery rates and increased level of satisfaction [26]. These advantages were especially evident in studies focusing on quality-of-life indicators, where OH was associated with improved post-procedural outcomes, particularly among patients undergoing evaluation for abnormal uterine bleeding, infertility, and postmenopausal bleeding. In contrast, ORH, though vital for complex pathology requiring extensive intervention, was linked with longer procedure durations, higher resource utilization, and increased reliance on anesthesia [27].

The narrative synthesis further highlights the diagnostic equivalence of OH in detecting common intrauterine abnormalities such as polyps, fibroids, adhesions, and endometrial hyperplasia. These results are especially relevant in the context of evolving healthcare models that emphasize outpatient care, patient autonomy, and cost containment. Several included studies emphasized the scalability of OH services, with implications for reducing surgical wait times and optimizing gynecologic care in both high-resource and resource-limited settings [28]. 

However, the review also identified moderate-to-high heterogeneity (I² > 70%) in some meta-analyses, particularly in procedural completion and complication rates. This variability likely reflects differences in operator experience, equipment availability (e.g., miniaturized hysteroscopic instruments), patient selection, procedural techniques, study design, and outcome reporting across the included literature. The predominance of observational studies, narrative reviews, and expert commentaries over randomized controlled trials may reduce the strength of the evidence. However, most studies reported short-term outcomes, with limited data on long-term recurrence, reproductive outcomes, and cost-effectiveness.

To conclude now, OH depicts itself as a very efficient and patient-friendly method of investigating and treating intrauterine pathology. It has significant clinical and logistical benefits against ORH, especially in undertaking simple diagnostic procedures and minor surgical procedures. A future study should be aimed at the standardization of procedures for selecting patients and management of pain, as well as an evaluation of the long-term results of the procedure, including rates of potential recurrence and effects on fertility. Also, multicenter randomized trials and cost-effectiveness analyses are needed to further empower the evidence base and promote the wider implementation of issues and widespread use of OH in various healthcare settings.

## Conclusions

This systematic review cements the notion that office hysteroscopy (OH) as a constituent part is a safe, efficacious, and patient-centered alternative to conventional orthodox operating room hysteroscopy (ORH) to diagnose and treat intrauterine pathologies. In a procedure with a high rate of completion, a low risk of complications, and positive pain and satisfaction ratings, OH becomes an efficient form of care that represents modern outpatient and minimally invasive care standards. ORH is still critical to complex interventions, but OH presents high accessibility, expenditure savings, and comfort to patients, especially in the case of diagnostic and minor surgical procedures. The data indicate that OH can be further introduced to the mainstream gynecological practice with appropriate selection of patients and expertise in the use of this procedure. The future direction should involve a standardization of protocol, training of operators, and multisite trials to strengthen the long-term sustainability and efficacy of OH benefits in a variety of clinical and resource-differing contexts.
